# Comparison of Oxford Nanopore Technologies and Illumina MiSeq sequencing with mock communities and agricultural soil

**DOI:** 10.1038/s41598-023-36101-8

**Published:** 2023-06-08

**Authors:** Bo Maxwell Stevens, Tim B. Creed, Catherine L. Reardon, Daniel K. Manter

**Affiliations:** 1 Water Management and Systems Research Unit, USDA ARS, Fort Collins, CO 80526 USA; 2 Soil Management and Sugar Beet Research Unit, USDA ARS, Fort Collins, CO 80526 USA; 3 Columbia Plateau Conservation Research Center, USDA ARS, Adams, OR 97810 USA

**Keywords:** Next-generation sequencing, Bacteria, Microbial communities, Next-generation sequencing

## Abstract

Illumina MiSeq is the current standard for characterizing microbial communities in soil. The newer alternative, Oxford Nanopore Technologies MinION sequencer, is quickly gaining popularity because of the low initial cost and longer sequence reads. However, the accuracy of MinION, per base, is much lower than MiSeq (95% versus 99.9%). The effects of this difference in base-calling accuracy on taxonomic and diversity estimates remains unclear. We compared the effects of platform, primers, and bioinformatics on mock community and agricultural soil samples using short MiSeq, and short and full-length MinION 16S rRNA amplicon sequencing. For all three methods, we found that taxonomic assignments of the mock community at both the genus and species level matched expectations with minimal deviation (genus: 80.9–90.5%; species: 70.9–85.2% Bray–Curtis similarity); however, the short MiSeq with error correction (DADA2) resulted in the correct estimate of mock community species richness and much lower alpha diversity for soils. Several filtering strategies were tested to improve these estimates with varying results. The sequencing platform also had a significant influence on the relative abundances of taxa with MiSeq resulting in significantly higher abundances Actinobacteria, Chloroflexi, and Gemmatimonadetes and lower abundances of Acidobacteria, Bacteroides, Firmicutes, Proteobacteria, and Verrucomicrobia compared to the MinION platform. When comparing agricultural soils from two different sites (Fort Collins, CO and Pendleton, OR), methods varied in the taxa identified as significantly different between sites. At all taxonomic levels, the full-length MinION method had the highest similarity to the short MiSeq method with DADA2 correction with 73.2%, 69.3%, 74.1%, 79.3%, 79.4%, and 82.28% of the taxa at the phyla, class, order, family, genus, and species levels, respectively, showing similar patterns in differences between the sites. In summary, although both platforms appear suitable for 16S rRNA microbial community composition, biases for different taxa may make the comparison between studies problematic; and even with a single study (i.e., comparing sites or treatments), the sequencing platform can influence the differentially abundant taxa identified.

## Introduction

The current standard for characterizing microbiomes is the Illumina MiSeq sequencing platform, which produces 16S rRNA reads up to 300 bp, and around 550 bp if forward and reverse reads are joined. Conversely, the Oxford Nanopore Technologies (ONT) MinION sequencer can potentially sequence more than 200,000 base pairs^1^. The main concern for using MinION sequencing is the lower base-calling accuracy, which is currently estimated around 95% compared to 99.9% for MiSeq^1^. However, continuous improvements are expected to improve accuracy substantially. The accuracy has increased to 96.5%, up from 65% when the MinION sequencer was first released^2^. Initial comparisons of these technologies indicate that MinION may be as good as, or better than MiSeq for taxonomic resolution at the genus and species level^3^. The accuracy of taxonomic assignment at the species level is considered to be low for both technologies^4^; however, the recent release of a new expectation–maximization algorithm-based classifier (Emu) may improve species level classification; particularly for full-length rRNA sequences^5^.

Bias and errors are introduced at many steps throughout the data production and analysis pipeline. Different DNA extraction methods and primer selection can have effects on the relative abundance of microbes^6,7^; high GC contents may reduce PCR efficiency^8^. PCR conditions such as annealing and denaturation time can have an impact on taxonomic output^9^. Furthermore, the reference database used for identification will also influence taxonomic assignments for both MiSeq and MinION^3^.

Bioinformatic methods are still in development for the MinION sequencer. Throughout the history of MiSeq sequencing, continuous improvements have been made to the bioinformatics pipeline, resulting in the removal of spurious sequences that artificially inflated estimates of alpha diversity^10–13^. Previously, sequences were clustered into operational taxonomic units (OTUs) using a similarity threshold (e.g., 99%), to minimize sequencing artifacts. Filtering thresholds based on abundance data have been used to remove rare OTUs that are typically associated with PCR and sequencing errors^14,15^. Currently, denoising techniques provide the best methods to estimate richness of microbial communities. QIIME2 with DADA2 provides the best estimate of richness, based on sequencing of complex mock communities^13,16^. Unfortunately, DADA2 is not available for MinION output^10^.

The purpose of this study was to compare the results of the MiSeq and MinION sequencers. Specifically, we wanted to examine if the sequencing platform influences: (1) estimates of species richness, (2) relative abundances of specific taxa, and (3) interpretations or comparisons between different sites. Because the typical protocol for each platform utilizes different sequencing primers and bioinformatics; we generated sequencing libraries using typical 16S rRNA protocols for each platform (i.e., full-length with MinION and V3–V4 region with MiSeq) and under conditions as similar as possible (e.g., V34 rRNA primers on both platforms).

## Methods

### Study sites

Soils were collected from two different sites (ARDEC: Colorado State University’s Agricultural Research, Development and Education Center in Fort Collins, CO; and CPCRC: USDA Columbia Plateau Conservation Research Center in Pendleton, OR). At each site, four replicate plots of no-till corn (ARDEC) or no-till annual wheat (CPCRC) were sampled. At ARDEC, the soils are clay loam and CPCRC the soils are Walla Walla silt loams (fine-loamy, mesic Aridic Haplustalls). For each plot, six 1″ diameter cores (15 cm deep) were sampled near plant crowns, composited in resealable plastic bags and stored on ice in coolers until transfer to the laboratory (less than 30 min). Once in the laboratory, the soils were homogenized by hand, sieved to 4 mm, and stored in the freezer (− 20 °C) until DNA extraction. Prior to freezing, subsamples (~ 5 g) were removed from each sample to measure gravimetric soil water content.

### DNA extraction

DNA was extracted from three replicate 0.25 g soil samples from each plot using the Qiagen DNeasy Powersoil Pro Kit (Qiagen, Germantown, MD). The extraction process was carried out using a fully automated Qiagen QIAcube robot with a 10-min vortex lysis step. DNA quality was assessed using a Nanodrop 1000 (Thermo Scientific, Waltham, MA) and quantified fluorometrically with the Invitrogen dsDNA HS Assay Kit on a Qubit 2.0 (Life Technologies, Carlsbad, CA).

### Library preparation

PCR amplifications were performed on each DNA sample using two different 16S rRNA gene primer pairs. The first primer pair, 341F/806R^17^, targets the V3-V4 region of the 16S gene and was used for both platforms. The second primer pair, 27F/1492R, targets the full-length 16S rRNA gene and was only used on the ONT MinION platform (Table 1).

**Table 1:** Summary of platforms and bioinformatics methods compared in this study.

Method	Platform	Adapter/Primer^1^	Target	Classifier	Error-correction
MinION V34	ONT MinION	341F: *TTTCTGTTGGTGCTGATATTGC* CCTACGGGNGGCWGCAG	V3–V4	minimap2	EMU
		806R: *ACTTGCCTGTCGCTCTATCTTC* GGACTACHVGGGTATCTAATCC			
MinION Full	ONT MinION	27F: *TTTCTGTTGGTGCTGATATTGC* AGRGTTYGATYMTGGCTCAG	Full-length	minimap2	EMU
		1492R: *ACTTGCCTGTCGCTCTATCTTC* TACCTTGTTACGACTT			
MiSeq V34	Illumina MiSeq	341F: *TCGTCGGCAGCGTCAGATGTGTATAAGAGACAG* CCTACGGGNGGCWGCAG	V3–V4	minimap2	EMU
		806R: *GTCTCGTGGGCTCGGAGATGTGTATAAGAGACAG* GGACTACHVGGGTATCTAATCC			
MiSeq V34 DADA2	Illumina MiSeq	341F: *TCGTCGGCAGCGTCAGATGTGTATAAGAGACAG* CCTACGGGNGGCWGCAG	V3–V4	minimap2	DADA2
		806R: *GTCTCGTGGGCTCGGAGATGTGTATAAGAGACAG* GGACTACHVGGGTATCTAATCC			

^1^Adapters are in italics; gene-specific primers are underlined.

### ONT MinION PCR conditions and library preparation

Extracted DNA samples were amplified in 60 µL PCR reactions containing 30 µL Phusion HSII (Thermo Scientific) master mix, 0.6 µL of each forward and reverse primer (10 µM concentration), 21.6 µL molecular grade H_2_O, and 6 µL soil DNA diluted 1:20 with nuclease-free water. Reactions were held at 98 °C for 30 s, with amplification proceeding for 25 cycles at 98 °C for 15 s, 50 °C for 15 s, and 72 °C for 60 s with a final extension at 72 °C for 5 min. The PCR products (PCR1) were purified using AMPure XP beads (Beckman Coulter, Indianapolis, IN).

Unique barcodes (EXP-PBC096, ONT, Oxford, UK) were added to both ends of the DNA fragments by PCR. These were 50 µL PCR reactions containing 25 µL Phusion HSII master mix, 19 µL H_2_O, 1 µL of forward/reverse barcodes, and 5 µL PCR1 product diluted 1:10 with nuclease-free water. Reactions were held at 98 °C for 30 s, with amplification proceeding for 15 cycles at 98 °C for 15 s, 62 °C for 15 s, and 72 °C for 60 s; a final extension at 72 °C for 5 min. The barcoded products of this PCR reaction were purified a second time using AMPure XP beads.

Barcoded amplicons from all samples were pooled and prepared for sequencing using the SQK-LSK109 Ligation Sequencing Kit (ONT). The library was loaded on a MinION flow cell FLO-MIN106D-R9 (ONT) per manufacturers’ protocol and sequencing was started with a runtime of 48 h and voltage of − 180 V. All libraries included no template (H_2_O-only) negative controls and a mock community (ZymoBIOMICS Microbial Community DNA Standard D6305; Zymo Research, Irvine CA).

### MiSeq PCR conditions and library preparation

Extracted DNA was amplified in triplicate, in 20 µL PCR reactions containing 10 µL Maxima SYBR-green (Thermo Scientific), 2 µL of each forward and reverse primer (10 μL concentration), 4 µL molecular grade H_2_O, and 2 µL soil DNA diluted 1:20 with nuclease-free water. Reactions were held at 95 °C for 5 min, with amplification proceeding for 28 cycles at 95 °C for 40 s, 55 °C for 120 s, and 72 °C for 60 s; a final extension at 72 °C for 7 min. Thermocycling was performed with a Roche 96 Lightcycler (Roche, Indianapolis, IN). The products of the triplicate PCR reactions were pooled and purified using AMPure XP beads.

Nextera XT barcode sequences (Illumina, San Diego, CA) were added to both ends of the DNA fragments by PCR using 50 µL PCR reactions containing 25 µL Maxima SYBR-green, 10 µL H_2_O, 5 µL of each forward and reverse barcode (5 µM concentration), and 5 µL of sample PCR1 product. Reactions were held at 95 °C for 3 min, with amplification proceeding for 8 cycles at 95 °C for 30 s, 55 °C for 30 s, and 72 °C for 30 s; a final extension at 72 °C for 5 min. The barcoded products of this PCR reaction were purified a second time using AMPure XP beads. Barcoded amplicons from all samples were pooled and sequenced on an Illumina MiSeq instrument at Colorado State University using an Illumina MiSeq v3 600-cycle Kit with 25% PhiX spike-in (Illumina).

### Bioinformatics and sequence processing

#### Emu MinION and Emu MiSeq

Sequences generated on the MinION platform were base-called and demultiplexed using Guppy v6.0.1 (ONT). Except were otherwise noted, default parameters were used. Sequences were filtered based on length (V34: 300–600 bp; Full: 1000–2000 bp) and a minimum q-score of 70 using Filtlong v0.2.1^18^ and Cutadapt v3.2^19^. Chimeras were filtered using vsearch^20^, and taxonomy was assigned with minimap2 v2.22^21^. Error-correcting was done with Emu v3.0.0^5^, using default parameters (–min-abundance = 0.0001, –N = 50, –K = 500 MB, –keep-counts = FALSE), which applies an expectation minimization algorithm to adjust taxonomic assignments using up to 50 sequence alignments per sequence read.

Paired forward and reverse MiSeq reads were joined using PEAR v0.9.8^22^. Sequences were then filtered based on length (V34: 300–600 bp) and a minimum quality score of 70 using Filtlong v0.2.1^18^ and Cutadapt v3.2^19^. Chimeras were filtered using vsearch UCHIME v2.13.3^20^, taxonomy was assigned with minimap2 and error-corrected with Emu v3.0.0^5^.

For DADA2^23^ MiSeq, all primers were removed from demultiplexed raw fastq files using Cutadapt v3.2^19^ and amplicon sequence variants were inferred using the default pipeline in DADA2. Each sequence variant was classified to the default NCBI-linked reference database available from the Emu v3.0.0 website (https://gitlab.com/treangenlab/emu) using minimap2 v2.22^21^ and the primary alignment for each sequence was chosen with SAMtools v1.9^24^ and used for taxonomic assignments. One phylum of bacteria has not been assigned a name, and is reported as “p_of_Bacteria.” All downstream data analyses were performed on taxonomic abundance tables following classification using the rank level(s) defined below.

### Data analysis

Total library sizes were as follows: MinION Full 1,695,436 total sequence reads with an average of 66,843 reads per sample; MinION V34 2,318,235 total sequence reads with an average of 96,730 reads per sample; and MiSeq V34 2,111,798 total sequence reads with an average of 83,345 reads per sample (Table 2). Therefore, prior to calculating alpha diversity (i.e., species richness) estimates all samples were rarefied to 50,000 reads. Principal Coordinates Analysis (PCoA) was performed using Bray–Curtis distances (BC) calculated from square root-transformed, genus-level relative abundances, and significant differences between platforms and/or sites were tested using adonis in the vegan package for R^25^. Figure 3 was constrained by both platform and site. Differential abundances were tested using either the DESeq2 package or Wilcoxon test in the metacodeR package using a false discovery rate < 0.05^26^.

**Table 2:** Summary data of sequencing results from the four platform and bioinformatics pipelines (MinION Full, MinION V34, MiSeq V34, and MiSeq V34 DADA2). Similarity was calculated with Bray–Curtis against the expected Zymo mock community. F statistics are shown for perMANOVA results for site differences (F Site) with each full dataset, and plot differences (F ARDEC and F Pendleton) for each site subset.subset (ARDEC and Pendleton).

Method	Reads	Mock reads	Mock genera	Mock species	Mock similarity (%)	F (Site)	F (ARDEC)	F (Pendleton)
MinION Full	16,95,435	55,747	18	75	85.2	134.0	2.54	5.14
MinION V34	23,18,234	1,16,705	90	284	74.4	109.0	2.20	3.67
MiSeq V34	21,11,798	91,138	48	145	73.6	151.0	2.90	6.88
MiSeq DADA2	NA	NA	8	8	80.9	77.5	1.78	4.50

## Results

**Figure 1:**
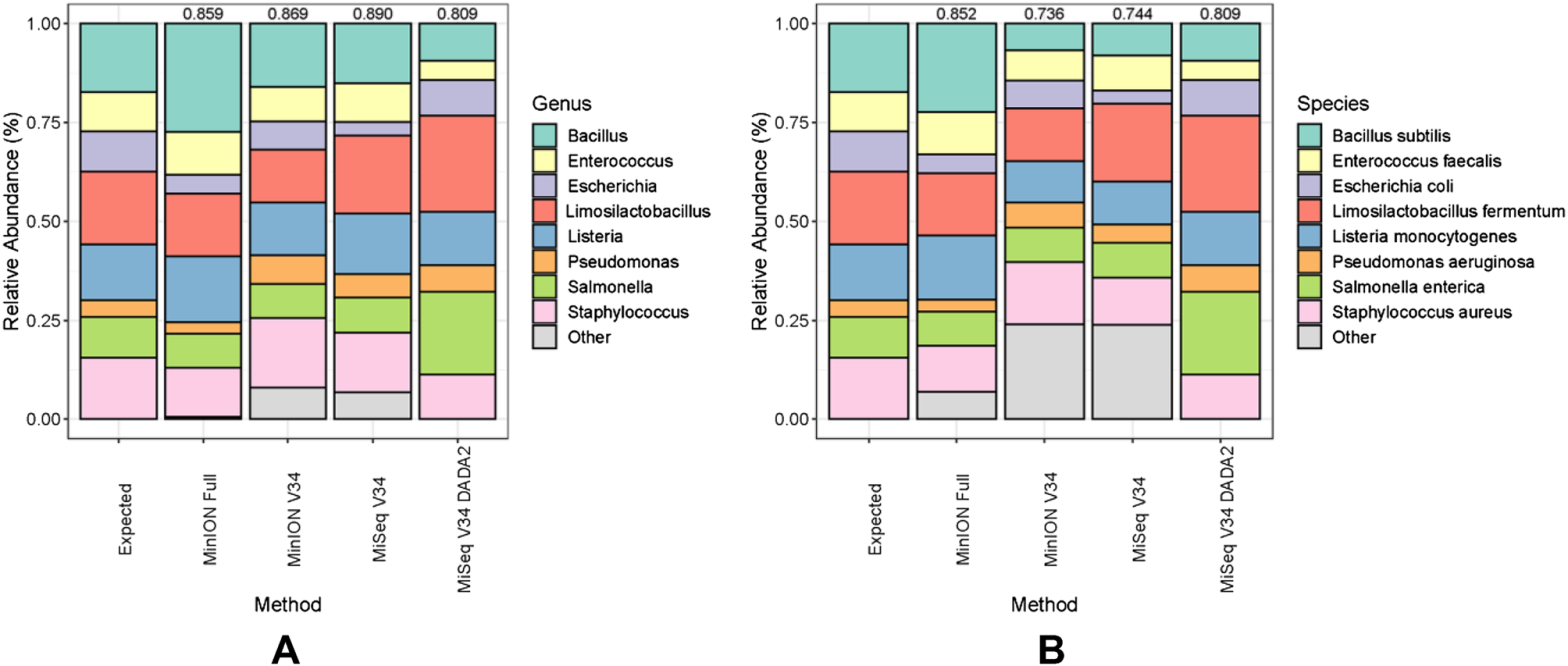
Taxonomic classification at the genus (**A**) and species (**B**) level for mock community sequencing output from the four platform and bioinformatics pipelines (MinION Full, MinION v34, MiSeq v34, and MiSeq V34 DADA2). Taxa not contained in the ZymoBIOMICS standard were grouped into the ‘Other’ category. Similarity (Bray–Curtis) between the expected mock community output and the sample is displayed above each sample.

Sequencing of the ZymoBIOMICS mock community standard for the MinION Full, MinION V34, and MiSeq V34 libraries yielded 55,747, 116,705, and 91,138 high-quality reads (Table 2). Sequences from these libraries were classified to 18, 90, 48, and 8 genera or 75, 284, 145, and 8 different species for the MinION Full, MinION V34, MiSeq V34, and MiSeq V34 DADA2 pipelines, respectively (Table 2). All eight of the expected ZymoBIOMICS bacterial species present in each sample. Despite the extraneous taxa (“Other”) observed in the MinION and MiSeq Emu pipelines, the highest Bray–Curtis similarity (1 − BC) to the mock community at the species level was observed for the MinION Full (0.852), followed by the MiSeq V34 DADA2 (0.809), MiSeq V34 (0.744), and MinION V34 (0.736) (Fig. 1 and Table 2).

**Figure 2:**
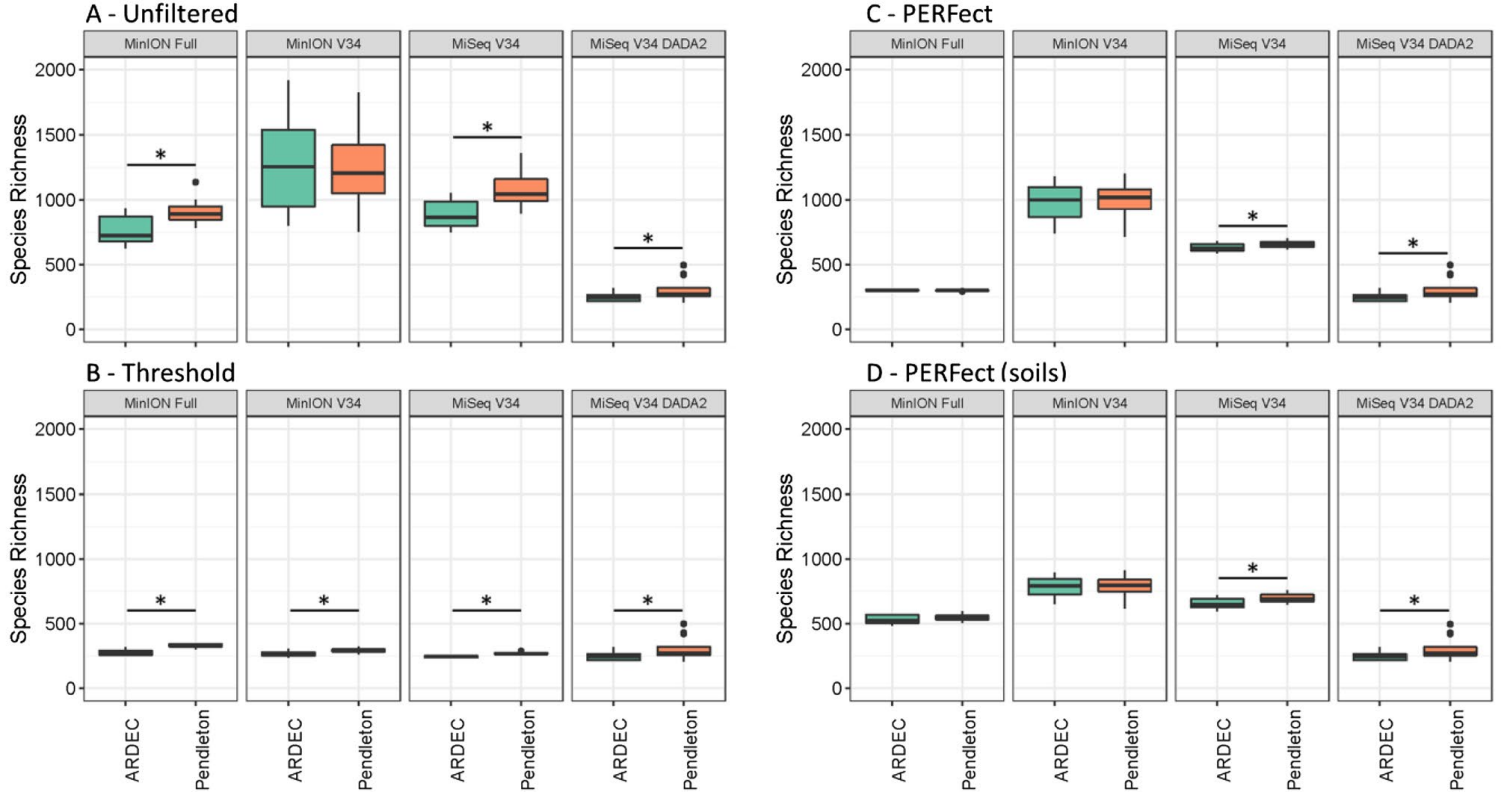
Species richness at two sites for all four sequencing methods and bioinformatics methods (MinION Full, MinION V34, MiSeq V34, and MiSeq V34 DADA2) for unfiltered (**A**), filtered at 0.07% relative abundance threshold (**B**) filtered by permutation using all samples (**C**), and filtered by permutation using only soil samples (**D**). Data were rarefied to 50,000 reads per sample and asterisks indicate a significant difference between sites (*p* ≤ 0.05). In all panels, the MinION V34 DADA2 method was calculated using unfiltered data only.

For the soil samples from two different agricultural sites (ARDEC, Colorado and Pendleton, Oregon), all methods except MinION V34 detected significant differences in species richness estimates; however, methods varied greatly in their richness estimates (Fig. 2A). For example, the MinION Full method estimated species richness of 762 for ARDEC and 909 for Pendleton with a *p*-value = 0.002. For the MinION V34, species richness estimates were 1276 for ARDEC and 1228 for Pendleton with a *p*-value = 0.748. The MiSeq V34 method resulted in 887 species for ARDEC and 1072 species for Pendleton (*p*-value = 0.001). Applying the DADA2 pipeline to this same library greatly reduced species richness: 248 species at ARDEC and 307 species at Pendleton (*p*-value = 0.047). Based on the inflated species richness in the non-DADA2 pipelines (> 8 species in the mock community and up to fivefold greater richness in the soil samples), we tested the effect of three different filtering methods on alpha diversity estimates in the non-DADA2 pipelines. The first method was to (1) remove all species below a user-specified relative abundance threshold, the second method employed a permutation-based strategy (PERFect R package, Smirnova et al. 2018) using either (2) all samples in each library (i.e., soils and mock communities) or (3) only the soil samples in each library. Iterative testing showed that a relative abundance threshold of 0.07% and 1% was necessary to achieve richness estimates that were closest to the MiSeq DADA2 levels for the soil and mock communities, respectively (Fig. S1). The threshold (0.07%) filtering approach resulted in similar species richness estimates between the filtered and non-filtered DADA2 pipelines with significant differences between sites (Fig. 2B); MinION Full: *p* < 0.001 (mean = 277 ARDEC; 331 Pendleton), MinION V34: *p* = 0.003 (266 ARDEC; 292 Pendleton), MiSeq V34: *p* < 0.001 (244 ARDEC; 270 Pendleton), and MiSeq V34 DADA2: *p* = 0.047 (247 ARDEC; 306 Pendleton). Both permutation methods significantly reduced alpha diversity measurements but still resulted in greater richness estimates than the unfiltered MiSeq V34 DADA2 pipeline (Fig. 2C,D); furthermore, significant site differences (p ≤ 0.05) were detected with the MiSeq but not the MinION pipelines.

**Figure 3:**
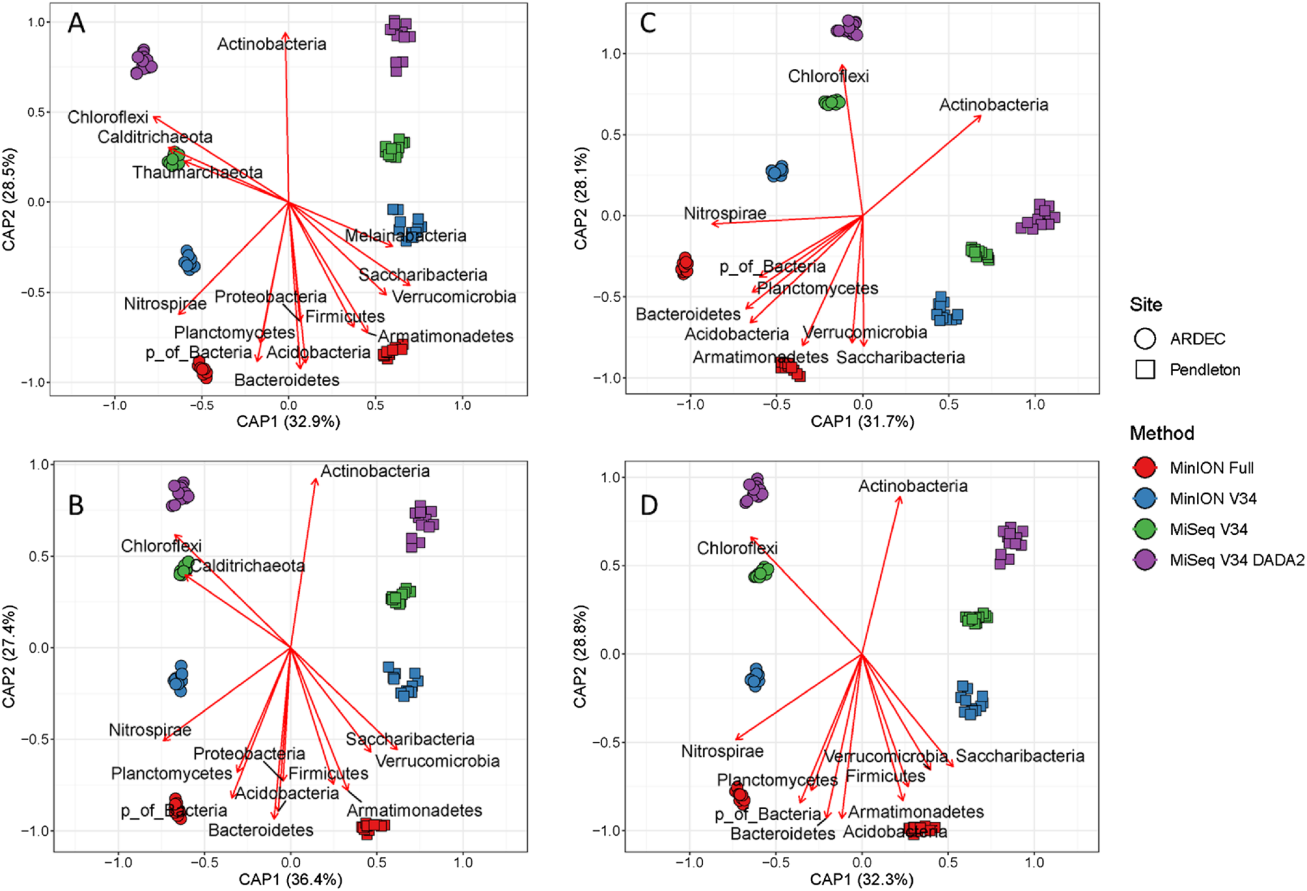
PCoA biplot of all four sequencing and bioinformatics methods (MinION Full, MinION V34, MiSeq V34, and MiSeq V34 DADA2) for unfiltered (**A**), filtered at 0.07% relative abundance threshold (**B**) filtered by permutation using all samples (**C**), and filtered by permutation using only soil samples (**D**). In all panels, the MinION V34 DADA2 method used unfiltered data only. Vectors indicate a significant correlation (*r*^2^ > 0.5, *p* < 0.01) between phyla relative abundance and ordination axes. CAP is a constrained axis.

**Figure 4:**
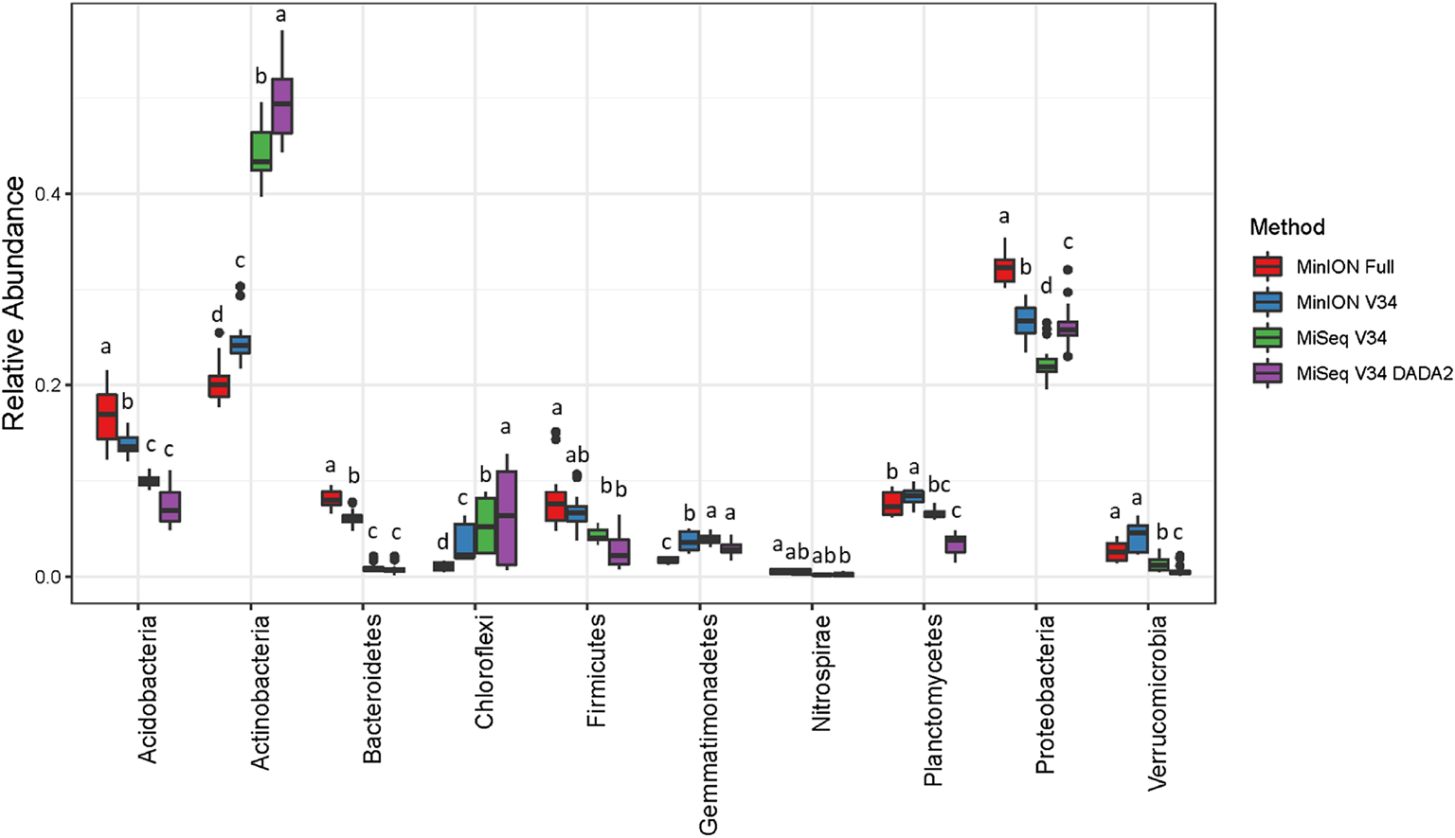
Relative abundance of the 10 most abundant phyla for all four sequencing bioinformatics methods (MinION Full, MinION V34, MiSeq V34, and MiSeq V34 DADA2). Bars with different letters are significant at an adjusted p ≤ 0.05 based on DESeq2 analysis with Benjamini–Hochberg correction. All data was unfiltered.

Genus-level community composition was significantly different between sequencing methods (F = 26.1, p = 0.001) and sites (F = 26.1, p = 0.001) based on a perMANOVA and visualized by PCoA (Fig. 3A) for the unfiltered data. The sites separated along Axis 1 (32.9%) and platforms (MinION vs MiSeq) separated along Axis 2 (28.5%). A biplot of phyla relative abundances showed that the MiSeq platform was enriched for Actinobacteria as compared to the MinION platform (Fig. 3A). Filtering had little impact on these patterns and perMANOVA showed that method and site differences (p = 0.001) were maintained (Fig. 3B–D). Biplots showed that phyla abundances showed similar patterns across all filtering methods with Actinobacteria positively correlated and Acidobacteria, Bacteroidetes, and Proteobacteria negative correlated with Axis 2 (r^2^ > 0.5). These patterns were confirmed by differential abundance analysis (DESeq2) which showed that the MiSeq platform (relative to the MinION) was enriched for Actinobactera, Chloroflexi, and Gemmatimonadetes; whereas, the MinION platform (relative to the MiSeq) was enriched for Acidobacteria, Bacteroidetes, Firmicutes, Proteobacteria, and Verrucomicrobia regardless of the bioinformatics pipeline used (Fig. 4). Filtering had no effect on the phyla detected as differentially abundant between platforms (data not shown). Differential abundance between sites was also tested using the non-parametric Wilcoxon test at all taxonomic levels from Kingdom to Family (Figs. S2–S4). Similar patterns were observed to the phyla level DESeq2 in which Actinobacteia, Gemmatimonadetes, and Chloroflexi were all enriched with the MiSeq platform. However, at finer taxonomic levels this phyla-level bias was not always consistent. For example, when comparing the MinION full to the MiSeq V34 DADA2 method, six sub-taxa of the Actinobacteria were lower with the MiSeq platform; consistent trends were also not seen with the taxa of the α, δ, and γ-Proteobacteria (Fig. S2).

**Figure 5:**
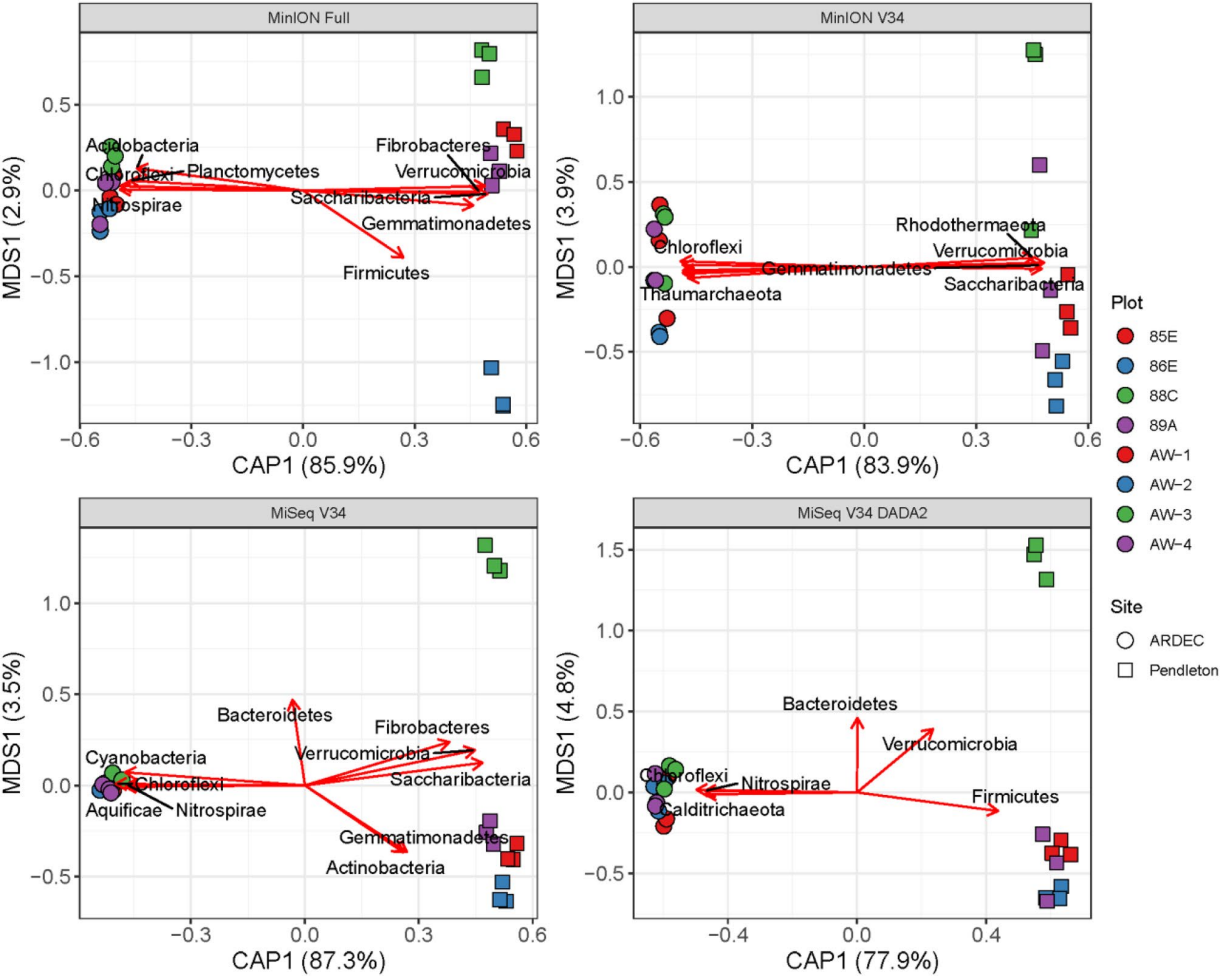
PCoA ordinations for each of the four sequencing and bioinformatics methods (MinION Full, MinION V34, MiSeq v34, and MiSeq DADA2). Shapes indicate different sites (ARDEC, Colorado and Pendleton, Oregon), while colors indicate separate plots within each site. Vectors indicate a significant correlation (*r*^2^ > 0.8, *p* < 0.01) between phyla relative abundance and ordination axes. The plot numbers in the legend are for internal reference and represent independent plot replicates under similar management at each site. CAP is a constrained axis and MDS is unconstrained.

At the plot level, all methods detected significant site and plot differences (Table 2). All *p*-values were less than 0.002 for all tests. Interestingly some of the patterns within a site differed; for example, at Pendleton, plot AW-2 is the most different for the MinION Full pipeline, whereas AW-3 is the most different for the MiSeq platform (Fig. 5). However, very little variation is explained by the second axis (2.9–4.7%).

**Figure 6:**
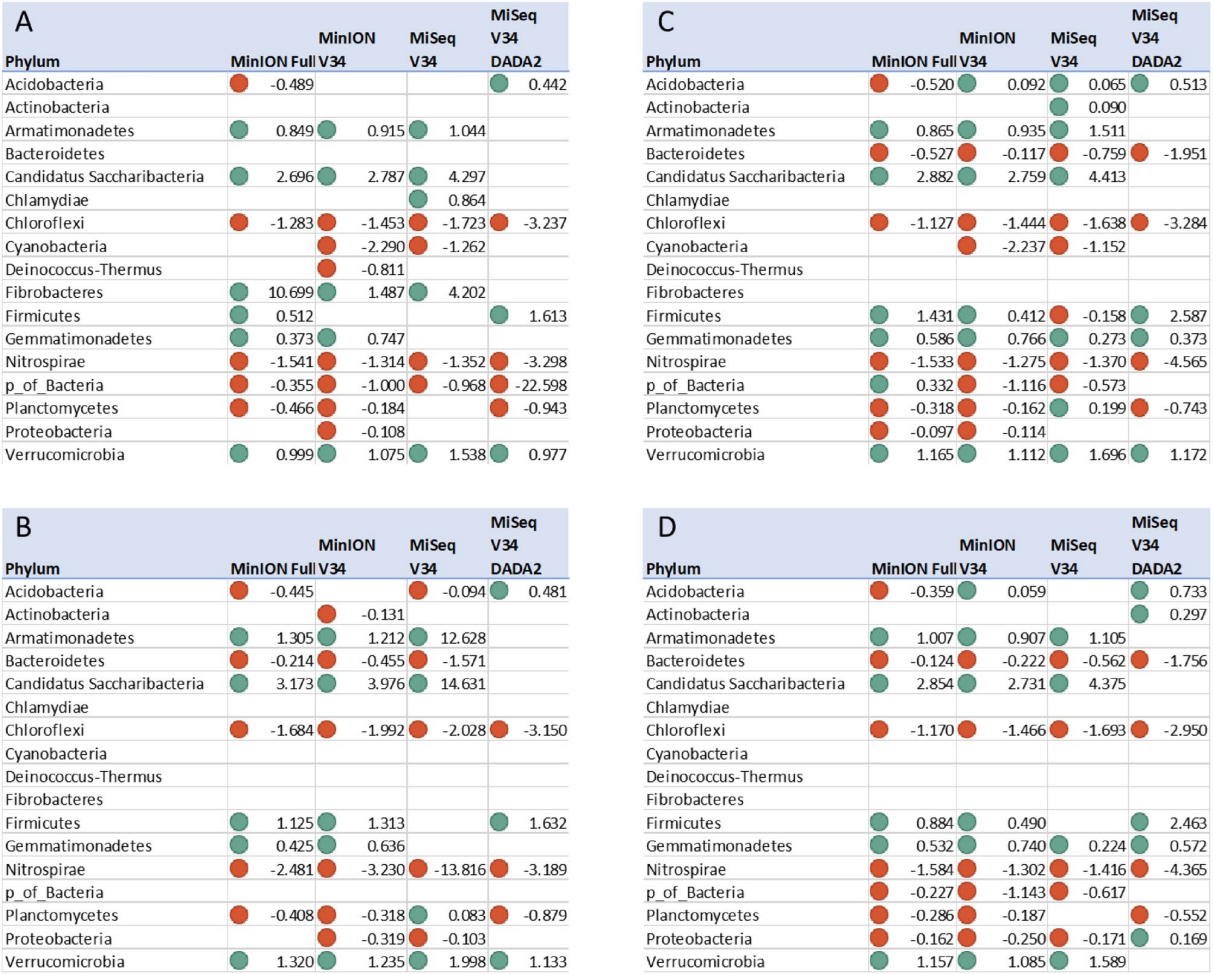
Log2 fold-change results from differential abundance (DESeq2) for each of the four sequencing and bioinformatics methods (MinION Full, MinION V34, MiSeq V34, and MiSeq DADA2). Each panel is a different filtering method: unfiltered (**A**), filtered at 0.07% relative abundance threshold (**B**), filtered by permutation using all samples (**C**), and filtered by permutation using only soil samples (**D**). Teal colors indicate significantly higher relative abundances in Pendleton, Oregon, while red indicates significantly higher abundances in ARDEC, Colorado. The lack of a point indicates that the test was not significant (false-discovery rate > 0.05).

**Figure 7:**
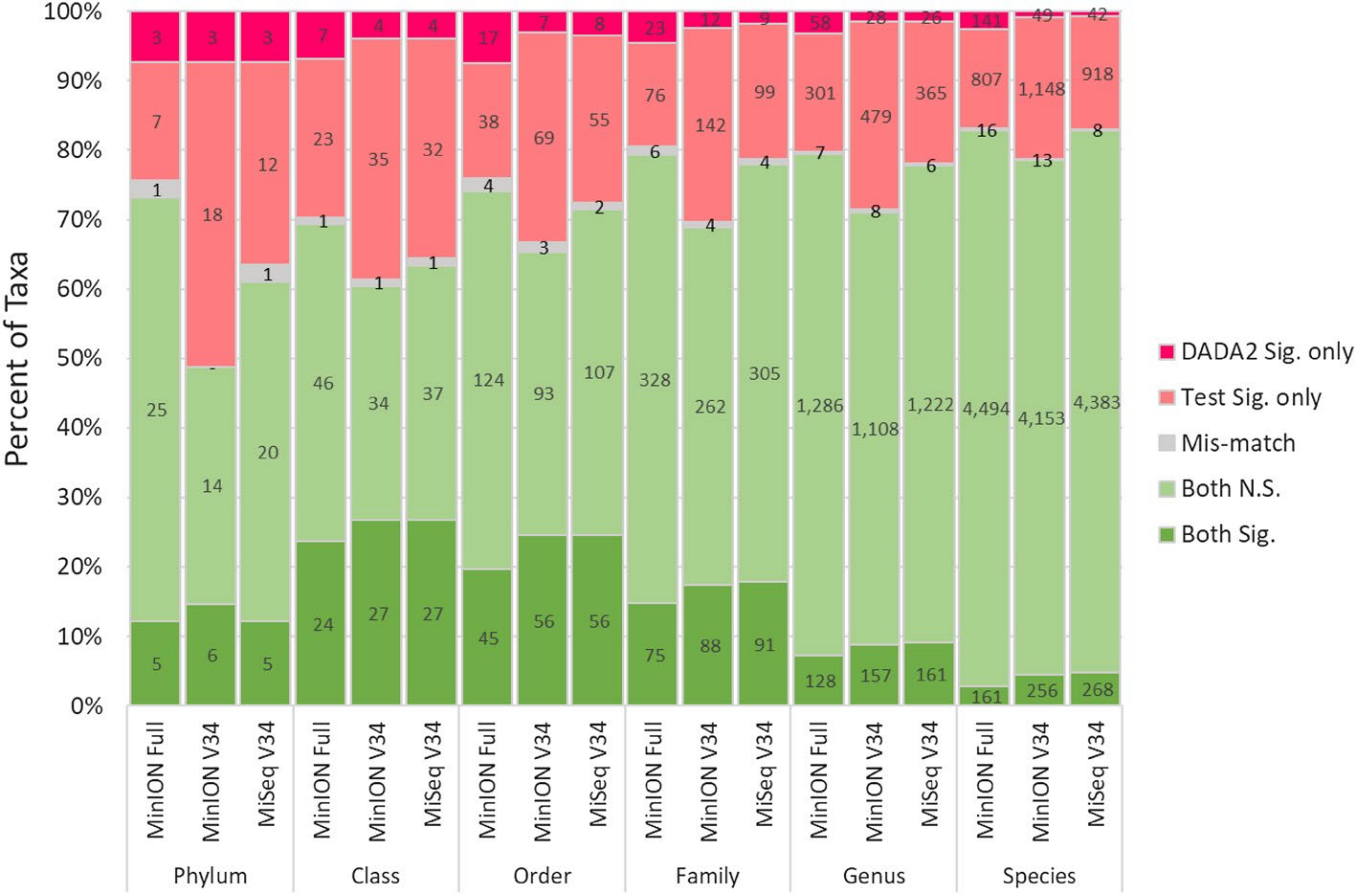
Percent of taxa significantly different between the two sites based on a Wilcoxon test of relative abundances. All methods are compared to the MiSeq V34 DADA2 pipeline where dark green bars (Both Sig.) are significantly different (FDR < 0.05) for both methods, light green bars (Both N.S.) are both not significantly different (FDR ≥ 0.05), grey bars (Mis-match) are significantly different (FDR < 0.05) but tests differ in enriched site, light red bars (Test Sig. only) are significantly different (FDR < 0.05) for only the test (i.e., listed) method, and dark red bars (DADA2 Sig. only) are significantly different (FDR < 0.05) for only the MiSeq V34 DADA2 pipeline. The numbers represent the total number of corresponding statistical tests of differential abundance between sites, in other words, taxonomic richness for each category.

Ideally, differential abundances of Phyla between the two soils should show the same magnitude and direction of change for all methods. However, this was not always the case within this study (Fig. 6). For instance, DESeq with unfiltered MinION Full data indicated that Acidobacteria was significantly more abundant in ARDEC, whereas MiSeq V34 DADA indicated Acidobacteria was higher in Pendleton, but both MinION V34 and MiSeq V34 showed no significant difference between sites (Fig. 6A). After removing species with less than 0.07% relative abundance, the differential abundance of Acidobacteria was similar between MiSeq V34 and MinION Full, but MinION V34 still indicated no significant difference (Fig. 6B). With some exceptions (e.g., Acidobacteria, Firmicutes, Planctomycetes, and Proteobacteria), all methods agreed on the direction of significant differences of phyla between sites. A more detailed analysis of differential abundance at all taxonomic levels was performed using the non-parametric Wilcoxon test of log2 median fold-changes, and results for the Kingdom through Family taxonomic levels were visualized with the metacodeR package (Figs. S5–S8). At all taxonomic levels, the full MinION method had the greatest agreement with the short MiSeq method with DADA2 correction compared to the other methods (Fig. 7). The two methods showed similar patterns in taxa between the two sites at the phyla (73.2%), class 69.3%), order (74.1%), family (79.3%), genus (79.4%), and species (82.3%) levels. Only a small fraction of taxa exhibited a mismatch between the two methods (i.e., significantly higher at opposite sites for each method).

## Discussion

Illumina MiSeq and the Oxford Nanopore Technologies MinION sequencing platforms have unique molecular methods for determining the sequence of DNA. Although MinION sequencing is a third-generation method with strong application in assessing microbial communities, it lacks the well-established bioinformatic methodology associated with second-generation MiSeq sequencing^13,16^. A previous comparison of dust microbial communities using these two sequencers suggested that there was generally good agreement between the two methods, with differences visible mainly at the genus and species taxonomic levels^3^. Up until now, comparisons of these two methods have not included mock communities along with complex agricultural soils, nor have they examined primer biases and different bioinformatics^3,4^. Our study adds to the previous studies by including a mock community, using similar primers on both platforms (V34 primers), and similar bioinformatics pipelines. Since the MiSeq platform with DADA2 error-correction is considered to be the current gold standard for estimating microbial community diversity^13^, we similarly used this method as our standard for comparison of MinION-generated data with full length and V3–V4 16S rDNA as well as MiSeq data analyzed with Emu.

### Species richness

The two platforms produce similar results with the low diversity, eight bacterial species mock community despite differences in the library preparation which were previously optimized for each platform. The MiSeq DADA2 pipeline resulted in no extraneous species; however, the MinION full-length method resulted in the closest similarity to the expected mock community composition. However, soil ecosystems have a much higher complexity of bacterial community composition with more than 40 phyla represented in a community versus the two phyla in the mock community. This complexity was captured to different degrees by the various methods tested here and some biases between methods were observed. In general, regardless of the platform, sequencing error-corrections algorithms or filtering methods appear to be necessary to remove extraneous DNA sequences and correct for over-estimates of alpha-diversity. The MiSeq with DADA2 correction consistently resulted in the lowest estimates of richness in soils compared to all other methods in this study.

Because sequencing methods produce experimental artifacts and inflate richness, we evaluated various filtering methods to remove potentially spurious taxa^13,15^. We tested both user-defined (relative abundance threshold) and permutation filtering methods to remove sequencing errors from soil samples. Assuming that the MiSeq DADA2 is the best estimate of soil bacteria richness, as it was for the mock community, only the relative abundance threshold method could result in similar species richness estimates for the other three methods. However, the required threshold appears to be dependent upon sample complexity (i.e., different thresholds for the soil and mock communities) and requires a user-defined threshold. In this study we were able to iteratively define the relative abundance threshold (0.07%) that resulted in similar estimates between methods; however, this will not always be possible because it is not feasible to sequence on both platforms for all future studies. This threshold will likely be dependent upon sequencing depth and in our case represents a much greater filtering threshold than just singletons or doubletons. For example, the 0.07% threshold requires a minimum read count of 47, 68, and 58 sequence reads per sample for the MinION full-length (66,843 average reads per sample), MinION V34 (96,730 average reads per sample), and MiSeq V34 (83,345 average reads per sample) methods. The impact of the permutation-based PERFect filtering method varied by method and complexity of the samples in the sequencing library. For example, permutation filtering in the MinION full-length was closest to the MiSeq DADA2 pipeline only when the controls (ZymoBIOMICS mock community and H_2_O controls) were included in the permutation filtering, whereas, the opposite was true for the MinION V34 pipeline. Diversity estimates for the MinION V34 and MiSeq V34 methods were more than three- and two-fold greater than the MiSeq V34 DADA2 pipeline regardless of the samples used in the permutation-based method.

### Relative abundance

One of the main goals of microbial community sequencing efforts is to also determine estimates of the abundance of specific taxa in the community. These abundances are frequently used as a proxy of microbial processes and soil functions^27^. Due to limitations in soil sample size and sequencing depths, comparisons are most frequently made using relative abundances or with normalized abundances. The relative abundance of phyla resulting from our various methods reveal that there are inherent biases in the sequencing platforms that can be seen at all taxonomic levels. For instance, MiSeq, regardless of bioinformatics method, tends to have a higher estimation of Actinobacteria and Bacteroidetes^28^. Both high and low GC contents can have a negative bias in the MiSeq platform^28^; however, as they suggest this is usually a problem in metagenomic sequencing and not rRNA sequencing where rRNA GC contents tends to fall within the optimal range (~ 50%). A quick analysis of the GC contents in the full-length rRNA reference database^5^ used here reveals an overall Phylum-specific mean of 55% with a minimum of 48% (Tenericutes) and maximum of 62% (Candidatus Bipolaricaulota). Early PCR termination is possible during amplification of GC-rich regions of the rRNA gene during library preparation^8^. However, all methods used here are reliant upon PCR amplification for library preparation and unless new biases are introduced during the MiSeq sequencing step (i.e., sequencing by synthesis) we suggest GC-biases are not likely to be the main driver of the platform differences. Furthermore, we did not see any systematic negative biases related to the GC content of ZymoBIOMICS mock community which was specifically designed to have a range of GC contents. For example, three of the species with > 50% GC content (*Salmonella enterica, Limosilactobacillus fermentum,* and *Pseudomonas aeruginosa*) all had higher, not lower, than expected frequencies with the MiSeq V34 DADA2 pipeline.

### Differential abundance

Another frequent use of microbial community data is to compare differences between locations and/or treatments for indicator taxa or changes in relative abundance. Ideally even if taxonomic abundances are biased, these biases would not interfere with the ability to identify relative differences between the treatments. Our results indicate that trends in differential abundance between soil sites were mostly consistent across sequencing methods with and without filtering; however, exceptions were observed at all taxonomic levels. Agreement between the site-level statistical difference was observed for over 70% of all taxa regardless of the method used. In previous studies it has been shown that differential abundance analyses are sensitive to sparsity (i.e., prevalence of samples with zero abundance)^29^ and do not always limit the detection of false-positives^30^. Furthermore, relative abundance differences are dependent upon microbial load or the total population present in a sample^31^. In this study, we compared sequencing analyses obtained from the same DNA extracts, so the different results should not arise due to different microbial loads.

## Conclusion

The MiSeq and MinION sequencing platforms both appear adequate for the assessment of microbial community composition. However, there are trade-offs worth considering which platform to use for a study: MiSeq offers a more established bioinformatics pipeline, while the MinION is capable of producing longer reads which may offer better assessments for fungal communities. While the cost per sample to sequence with each platform in our study was not much different, the barrier-to-entry for new labs may be an incentive for procuring a MinION sequencer. Also, depending upon the diversity of the sample being studied, conflicting results for relative abundances and alpha- and beta-diversity may arise. Without denoising algorithms like DADA2, estimates of richness from MinION sequencing will be inflated. Large differences in relative abundances of taxa between the sequencing and bioinformatics methods indicate we may need to be skeptical about relative abundance differences between studies, especially those with small trends. Overall, however, all methods were highly successful in identifying statistical differences of relative abundance between sites and more than 70% of the taxa showed agreement with the industry standard MiSeq DADA2, with full-length MinION sequencing resulting in the highest agreement. Additional studies are needed to identify if this variability is different than what would arise in multiple libraries generated within a single laboratory using consistent methods or between laboratories. As demonstrated here, the use of mock communities is critical for assessments but differences in the complexity limit our ability to make inferences about soil communities. Although more complex microbial reference standards are emerging, soil reference material is greatly needed and crucial in our ability to conduct meta-analyses in a field where technological changes occur at a rapid pace.

## Supplementary Information

Supplementary Figures.

## Data Availability

All raw sequence data in this study are available in NCBI under the Sequence Read Archive (SRA) BioProject ID PRJNA862376 (https://www.ncbi.nlm.nih.gov/bioproject/PRJNA862376/).
