# Supplementary material for: Comparison of Oxford Nanopore Technologies and Illumina MiSeq sequencing with mock communities and agricultural soil
Source: Sci Rep. 2023 Jun 8;13:9323. doi: 10.1038/s41598-023-36101-8 (PMC10250467; doi:10.1038/s41598-023-36101-8)
Supplement: Supplementary file 1 — Supplementary Figures. [file 41598_2023_36101_MOESM1_ESM.docx]

# Supplementary Figures


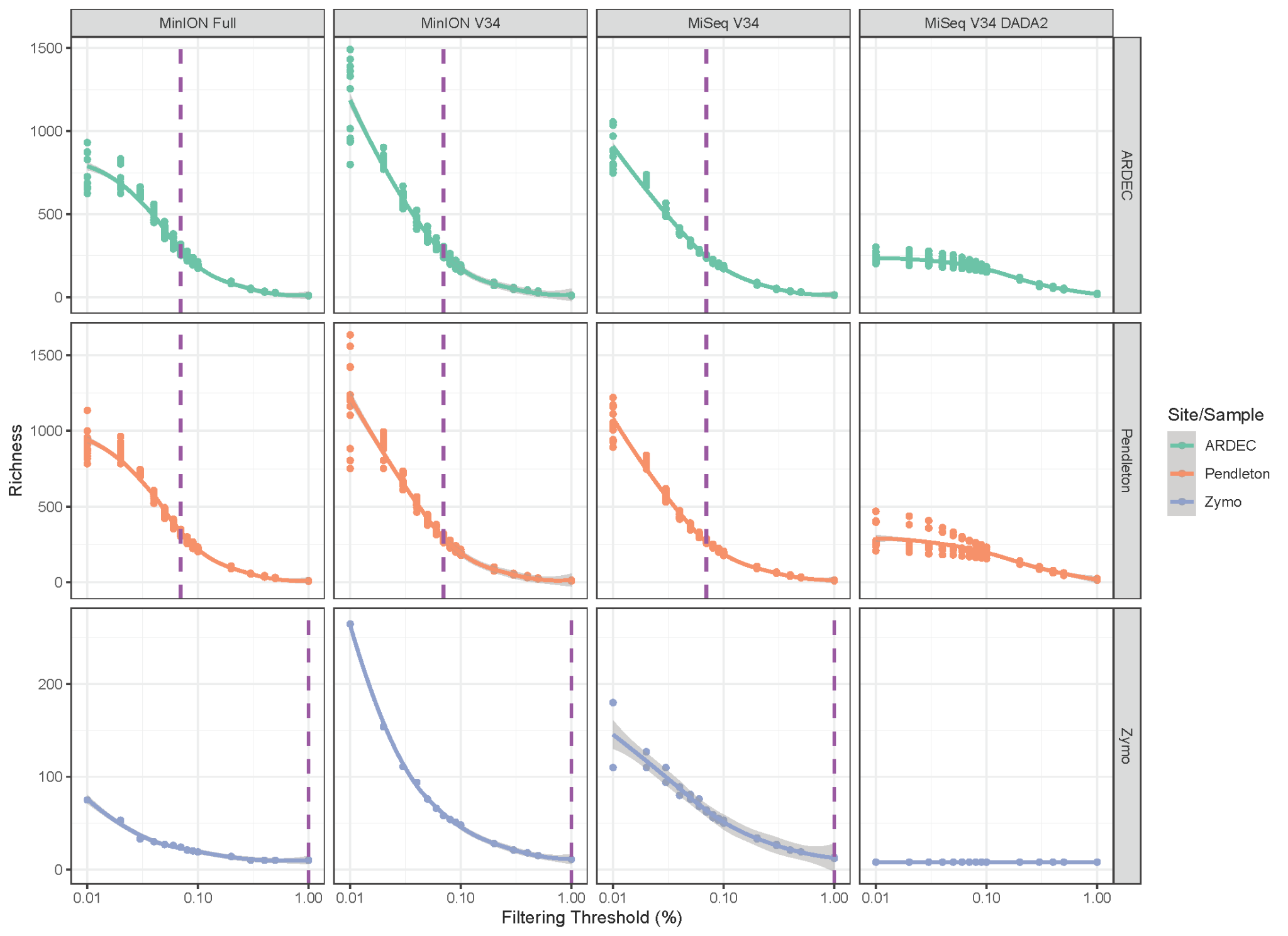


Figure S1. Richness estimates at various filtering thresholds for each of the four sequencing and bioinformatics methods (MinION Full, MinION v34, MiSeq v34, and MiSeq V34 DADA2). Purple dashed line indicates the threshold that is closest to the unfiltered richness estimate from the DADA2 pipeline.


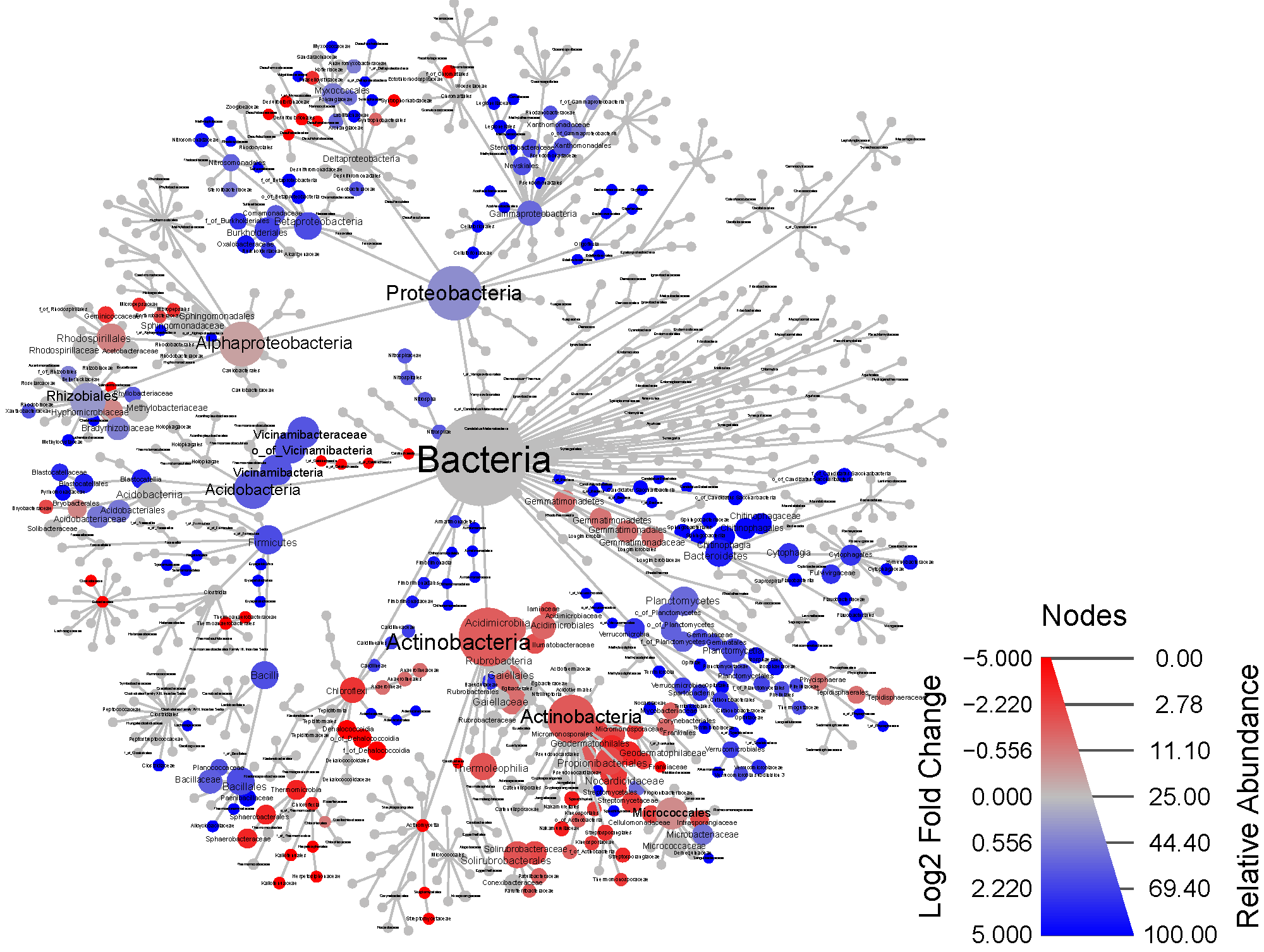


Figure S2. Taxonomic tree comparing the MinION Full vs MiSeq V34 DADA2 methods. Node size is the study-wide average relative abundance. Node color is the log2 median ratio comparing the ARDEC and Pendleton sites, non-significant Wilcoxon tests (FDR > 0.05) are shown in grey. Red indicates a lower abundance; blue indicates a higher abundance. Each node represents a taxonomic level from Kingdom to Family. Figure was produced with metacodeR.
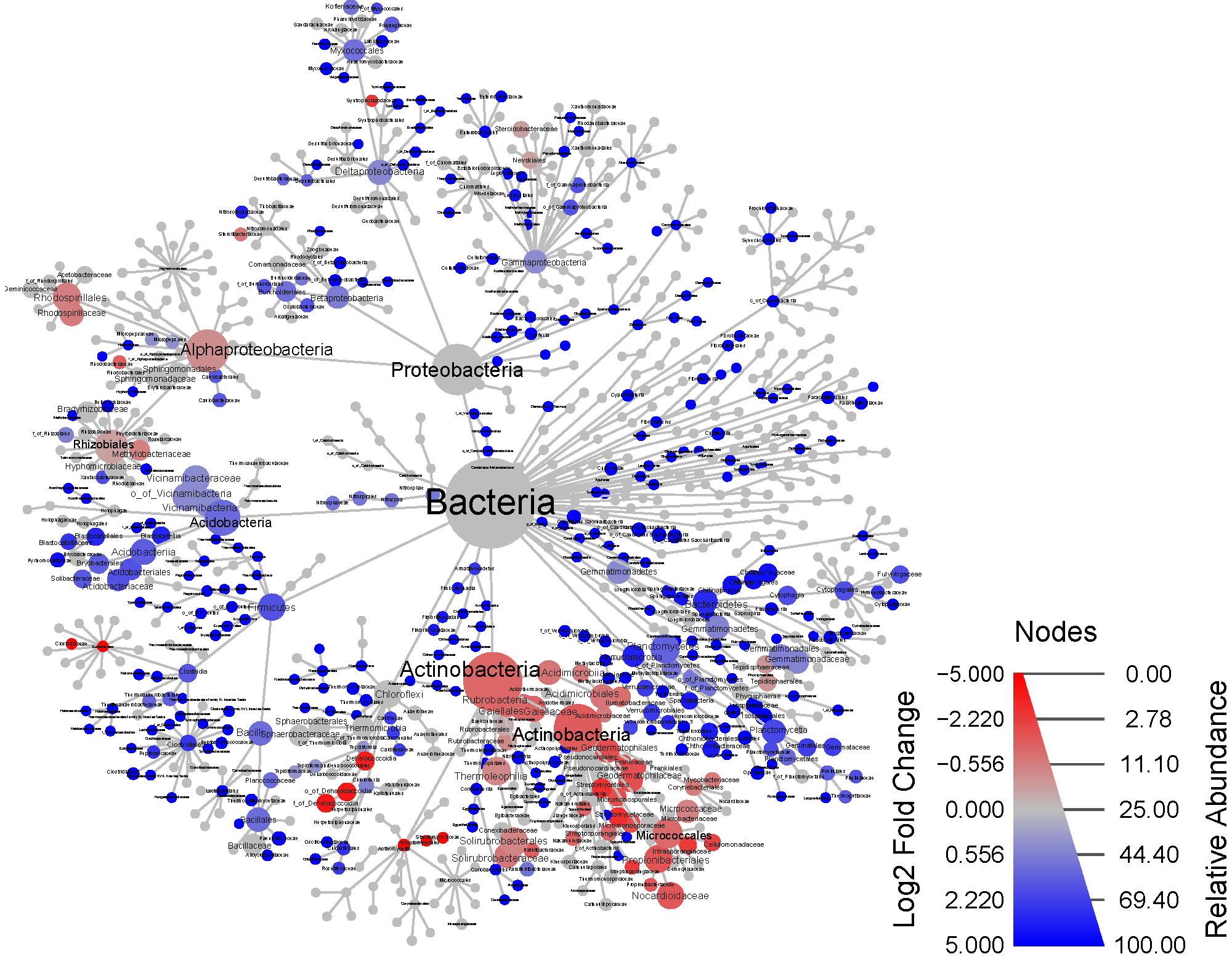


Figure S3. Taxonomic tree comparing the MinION V34 vs MiSeq V34 DADA2 methods. Node size is the study-wide average relative abundance. Node color is the log2 median ratio comparing the ARDEC and Pendleton sites, non-significant Wilcoxon tests (FDR > 0.05) are shown in grey. Red indicates a lower abundance; blue indicates a higher abundance. Each node represents a taxonomic level from Kingdom to Family. Figure was produced with metacodeR.


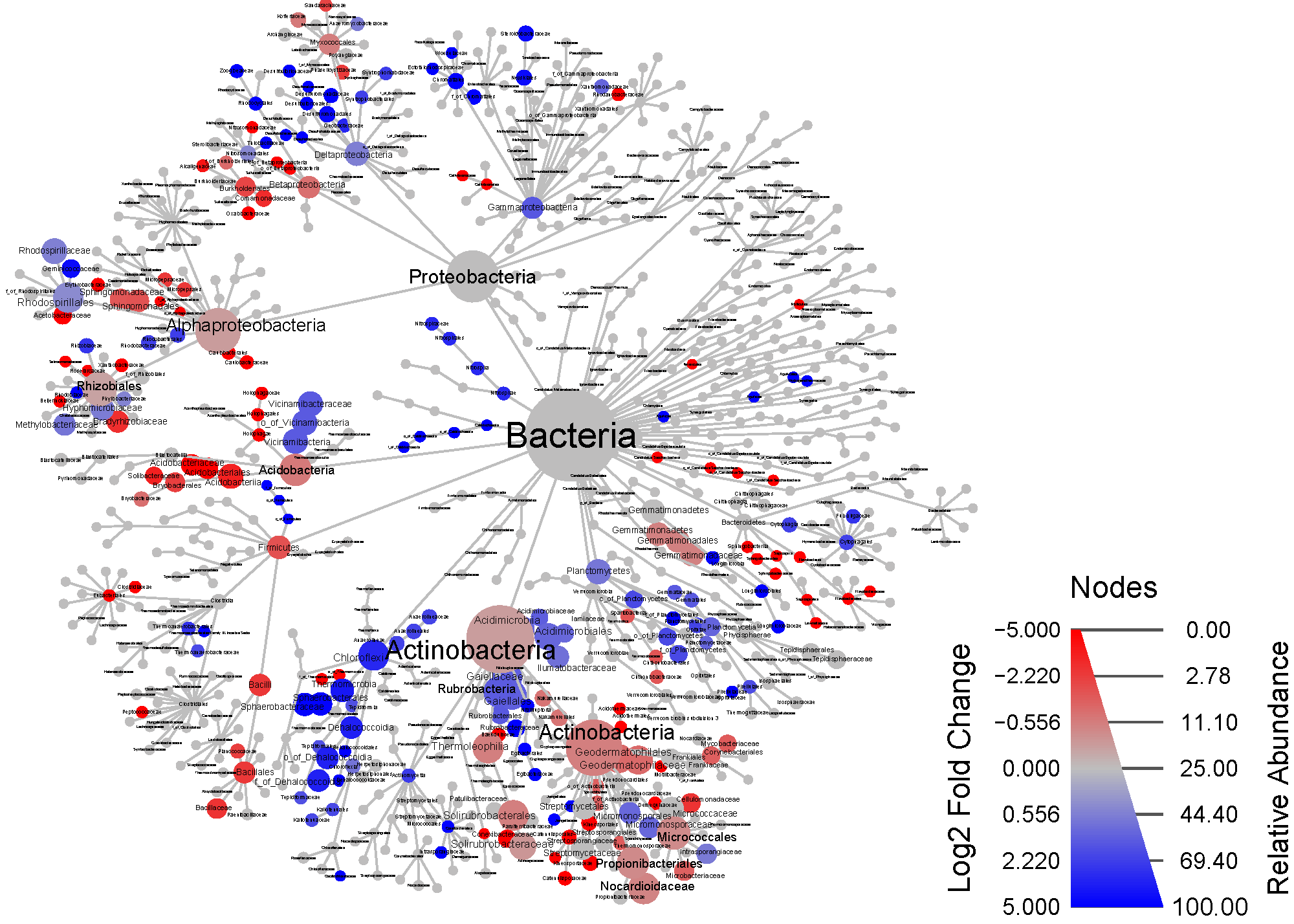


Figure S4. Taxonomic tree comparing the MiSeq V34 vs MiSeq V34 DADA2 methods. Node size is the study-wide average relative abundance. Node color is the log2 median ratio comparing the ARDEC and Pendleton sites, non-significant Wilcoxon tests (FDR > 0.05) are shown in grey. Red indicates a lower abundance; blue indicates a higher abundance. Each node represents a taxonomic level from Kingdom to Family. Figure was produced with metacodeR.


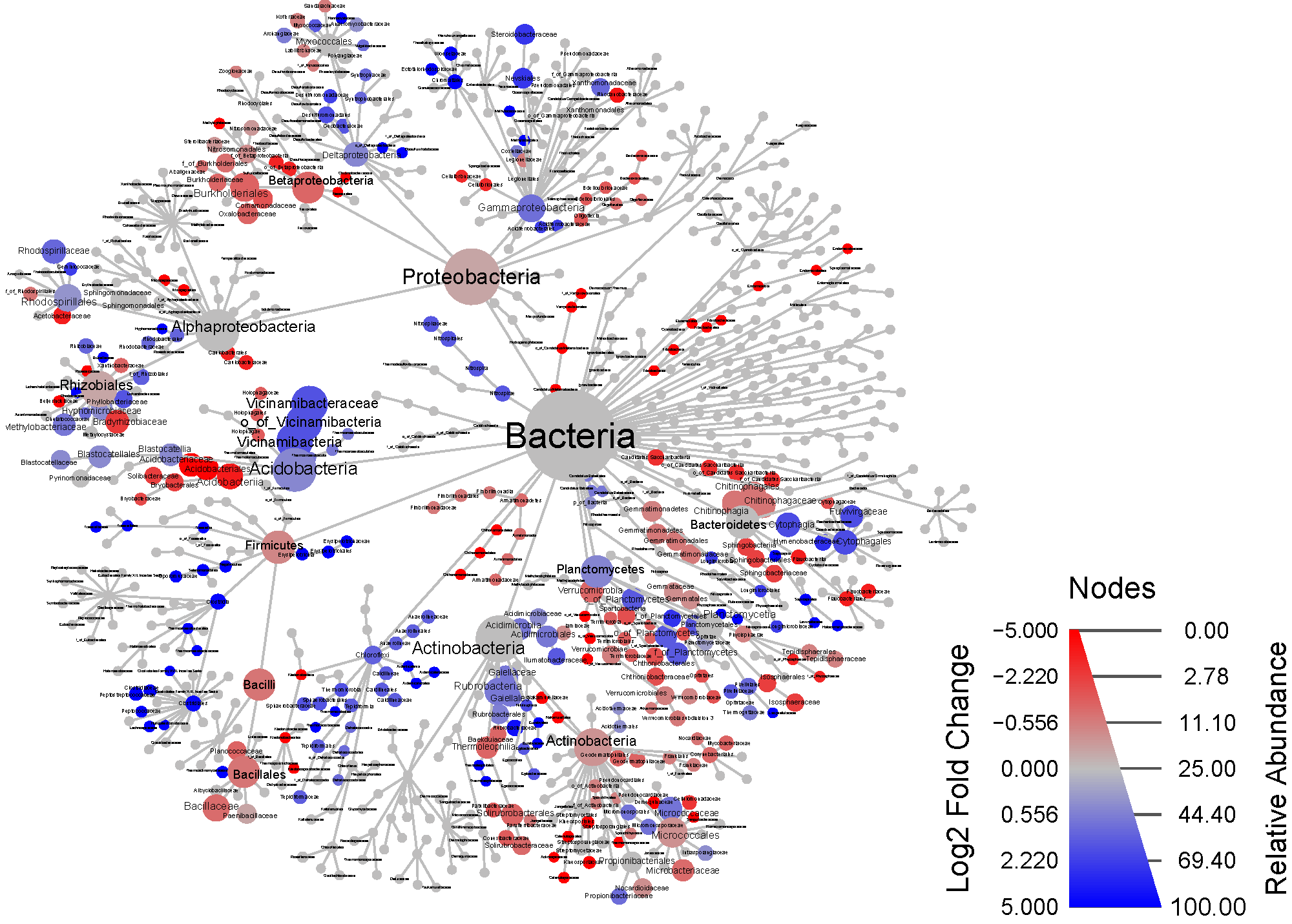


Figure S5. Taxonomic tree comparing site differences with the MinION Full method. Node size is the study-wide average relative abundance. Node color is the log2 median ratio comparing the ARDEC and Pendleton sites, non-significant Wilcoxon tests (FDR > 0.05) are shown in grey. Red indicates a lower abundance; blue indicates a higher abundance. Each node represents a taxonomic level from Kingdom to Family. Figure was produced with metacodeR.


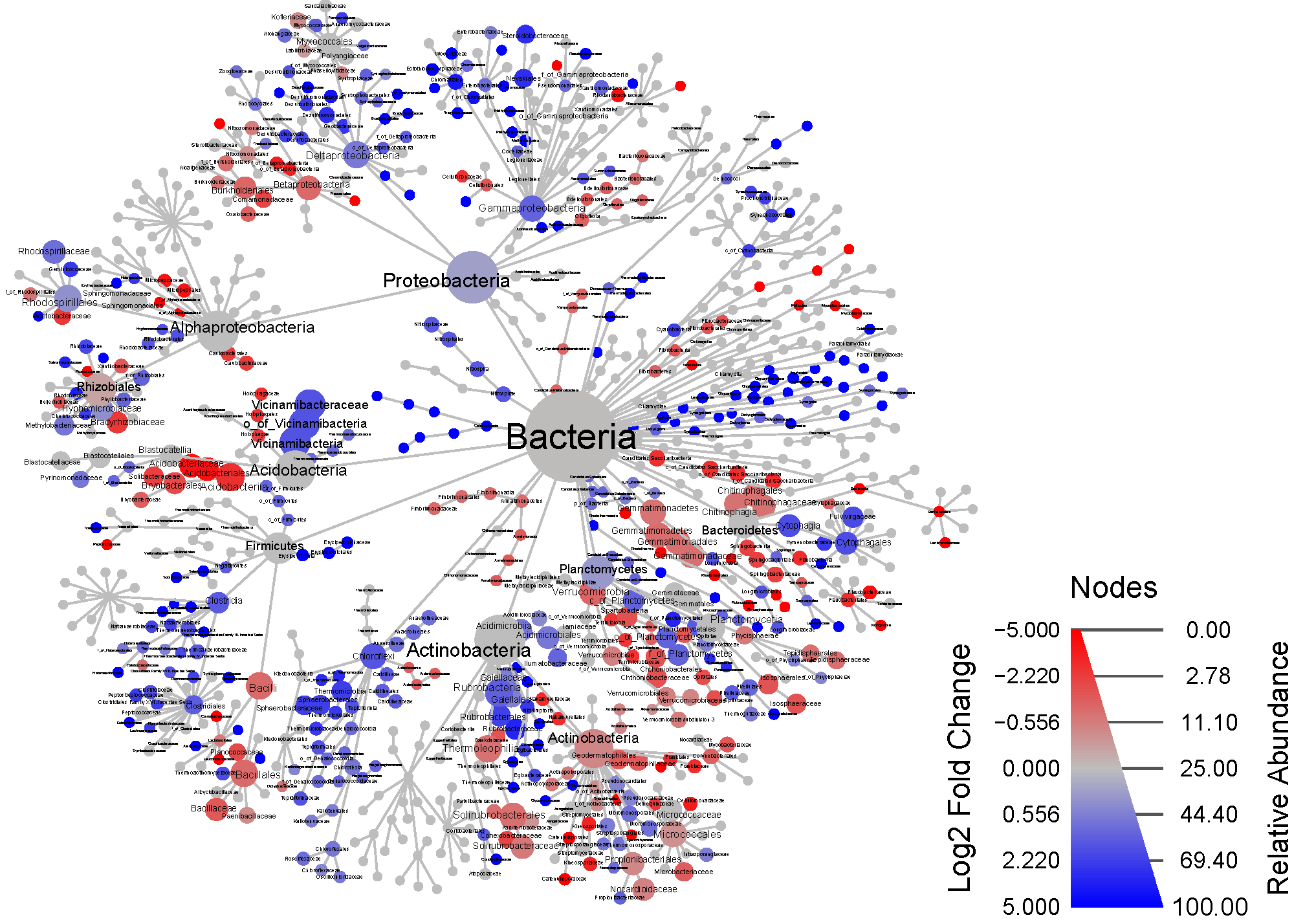


Figure S6. Taxonomic tree comparing site differences with the MinION V34 method. Node size is the study-wide average relative abundance. Node color is the log2 median ratio comparing the ARDEC and Pendleton sites, non-significant Wilcoxon tests (FDR > 0.05) are shown in grey. Red indicates a lower abundance; blue indicates a higher abundance. Each node represents a taxonomic level from Kingdom to Family. Figure was produced with metacodeR.


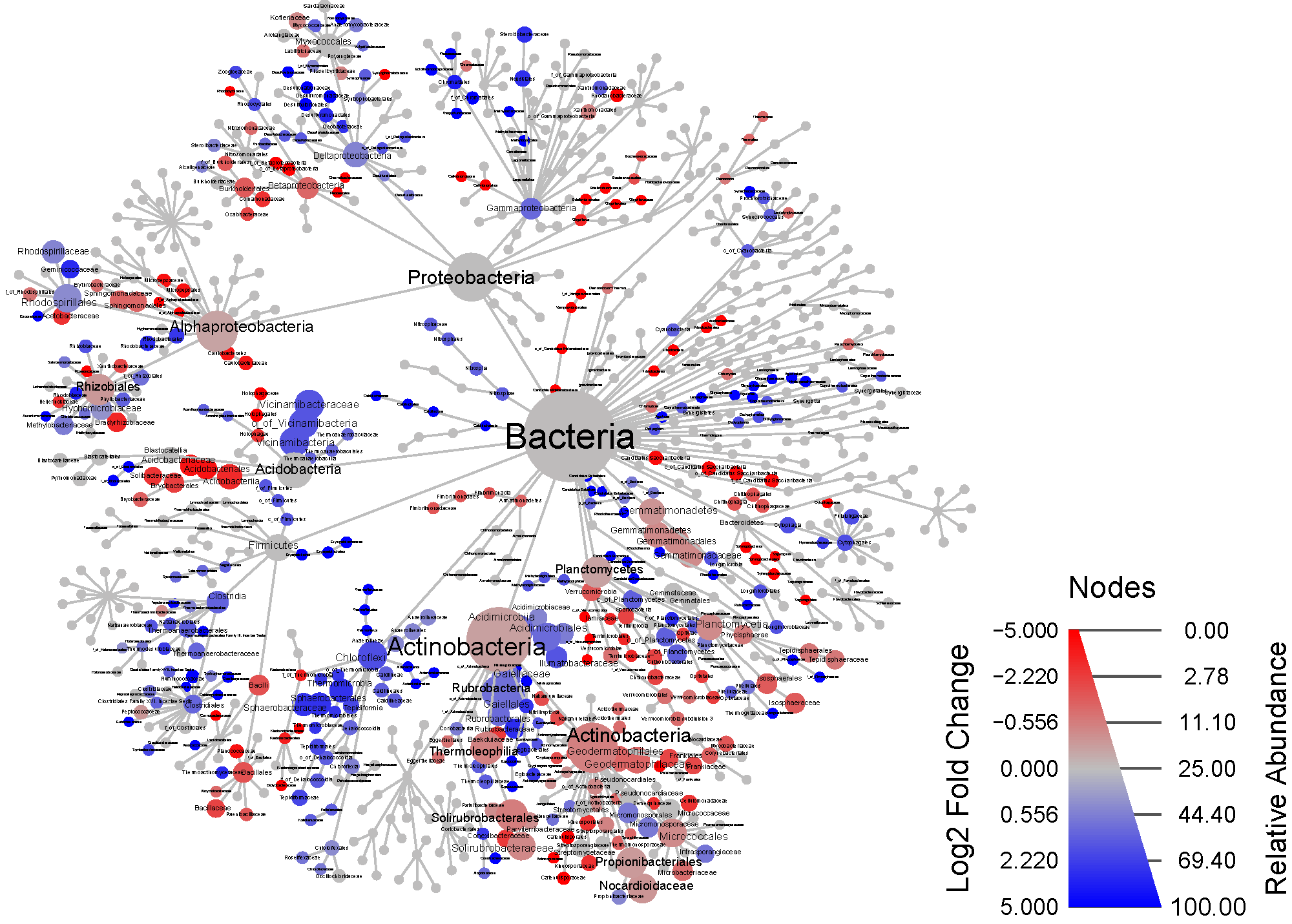


Figure S7. Taxonomic tree comparing site differences with the MiSeq V34 method. Node size is the study-wide average relative abundance. Node color is the log2 median ratio comparing the ARDEC and Pendleton sites, non-significant Wilcoxon tests (FDR > 0.05) are shown in grey. Red indicates a lower abundance; blue indicates a higher abundance. Each node represents a taxonomic level from Kingdom to Family. Figure was produced with metacodeR.


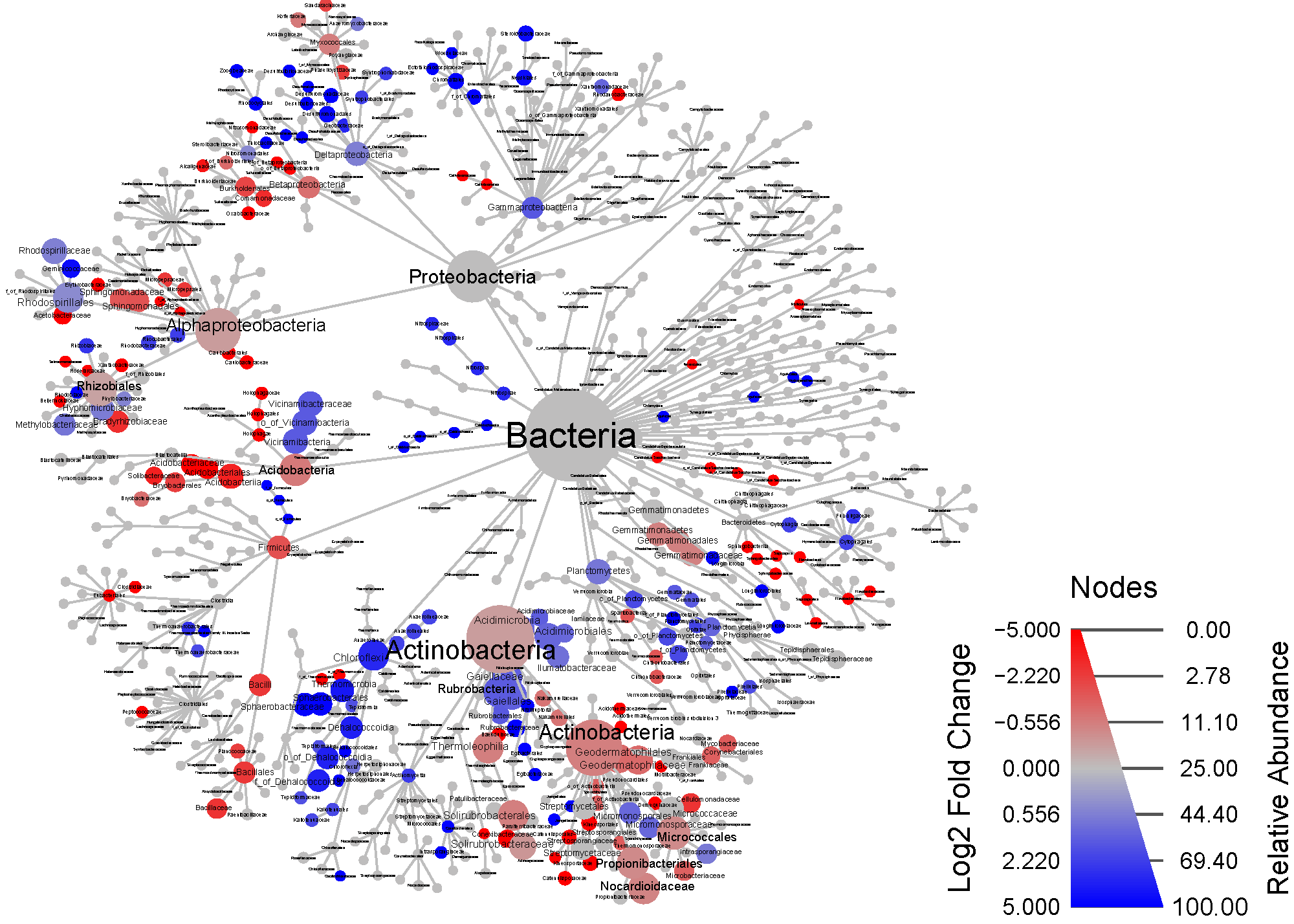


Figure S8. Taxonomic tree comparing site differences with the MiSeq V34 DADA2 method. Node size is the study-wide average relative abundance. Node color is the log2 median ratio comparing the ARDEC and Pendleton sites, non-significant Wilcoxon tests (FDR > 0.05) are shown in grey. Red indicates a lower abundance; blue indicates a higher abundance. Each node represents a taxonomic level from Kingdom to Family. Figure was produced with metacodeR.
